# Investigation of the Impact of Thionine Functionalization on Magnetoelastic Sensor Performance

**DOI:** 10.1021/acsabm.4c01488

**Published:** 2025-01-29

**Authors:** Wenderson R. F. da Silva, Gilberto Rodrigues-Junior, Eduardo N. D. de Araújo, Everton Pereira-Andrade, Ângelo Malachias, Joaquim B. S. Mendes

**Affiliations:** † Departamento de Física, Universidade Federal de Viçosa, Viçosa, Minas Gerais 36570-900, Brasil; ‡ Departamento de Física, Universidade Federal de Minas Gerais, Belo Horizonte, Minas Gerais 31270-901, Brasil

**Keywords:** magnetoelastic sensors, surface analysis, molecular adsorption, molecular functionalization

## Abstract

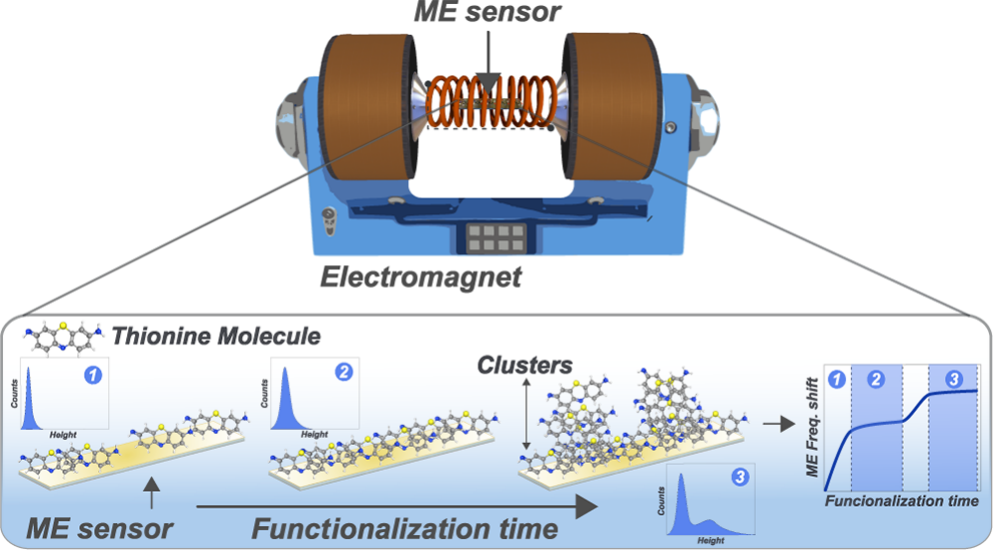

This study investigates the functionalization of gold-coated magnetoelastic sensors with thionine molecules, focusing on resonance frequency shifts. The functionalization process was characterized by using Raman spectroscopy and analyzed via scanning electron microscopy and atomic force microscopy, revealing the progressive formation of molecular clusters over time. Our results demonstrate that longer functionalization time leads to saturation of surface coverage and cluster formation, impacting the sensor’s resonance frequency shifts. Such modifications offer insights into the adsorption process of these molecules on the sensor’s gold surface, as well as its performance.

## Introduction

Magnetoelastic (ME) materials are recognized for their great potential in creating sensors with high sensitivity and selectivity for the detection of various physical, chemical, and biological agents due to their ability to detect very low (nanograms or lower) amounts of targets, as demonstrated by recent research.^1−3^ The suitable selection of the target to be detected is essential, highlighting the fundamental importance of the functionalization process to optimize the sensor performance. Sensor surface functionalization is a modification process to improve its detection properties or adapt it to a particular application.^4,5^ This process is done by binding molecules or chemical groups to the surface of the sensor, allowing a selective interaction with the target to be detected, thus increasing its sensitivity and selectivity, which is essential for the production of ME sensors and can directly affect their detection performance.^6^ Therefore, a detailed analysis of the functionalization process, exploring how the parameters linked to the adhesion of the functionalized substance to the sensor base, besides their implications on morphology, coverage, and roughness, is crucial and can contribute to improving the ME sensor’s performance.

Previous studies on functionalized ME sensors have used techniques such as atomic force microscopy (AFM), scanning electron microscopy (SEM), and energy dispersive X-ray spectroscopy (EDX) to investigate the adhesion of the functionalizing substance to its surface.^7−9^ The Kelvin probe force microscopy (KPFM) technique can contribute to a more improved analysis of the functionalization process, as it reveals binding and coverage patterns that are not observed in the traditional AFM method.^10^ By applying a bias voltage to the probe and measuring the resulting electrostatic force, the KPFM technique allows surface potential mapping across a sample surface, providing insights into its electronic properties, surface charge distribution, and surface potential gradients.^11,12^ These measurements provide additional information about the interaction between the functionalizing substance and the ME sensor substrate, and can significantly contribute to the understanding and optimization of the functionalization process.

On the other hand, the thionine molecule plays a crucial role as a linker in various sensor applications,^13,14^ facilitating surface functionalization and modification, including two-dimensional materials such as graphene and transition metal dichalcogenides.^15−19^ In this study, we examined the adsorption process of the thionine molecule onto the gold surface previously deposited on an ME sensor by monitoring the ME resonance frequency. The KPFM technique was used to simultaneously monitor the surface morphology and surface potential properties of the ME sensor at regular functionalization time intervals. Additional analyses using SEM and Raman spectroscopy were performed to confirm the functionalization and coating of the sensor surface. Finally, a comparison between the ME resonance frequency displacement curve and the measured surface parameters was performed with the aim of understanding the deposition dynamics recorded by the ME sensor.

## Materials and Methods

### ME Sensor Preparation

Magnetoelastic strip-shaped resonator platforms of 1 × 5 × 28 μm in size were fabricated from the METGLAS 2826MB3 alloy (Fe_40_Ni_38_Mo_4_B_18_). The ribbons were diced into rectangular-shaped platforms using scissors and a paper millimeter, which allowed precise cuts. The sensor was ultrasonically cleaned, first in acetone for 5 min, and then in ethanol for 5 min. Subsequently, the polished side of the sensor received a 50-nm thick layer of chromium (Cr) deposited by the magnetron sputtering technique. Later, they were coated with a 100-nm thick layer of gold (Au (111)) using thermal evaporation (see Figure S1). In these stages, an ATC Polaris 5 Sputter system and an Edwards Auto306 Turbo Evaporator equipped with an Edwards FTM6 quartz crystal microbalance to determine film thickness were used. The chromium (Cr) layer enhances adhesion between the gold (Au) film and the substrate, while the gold layer provides a highly biocompatible surface, promoting increased adsorption of functionalized biomolecules.^20^ Afterward, the ME ribbons were annealed in a vacuum (∼10^–3^ Torr) furnace at 200 °C for 2 h to relieve residual internal stress and promote adhesion of the Au layer to the ME ribbons. Subsequently, the ME platforms were used for the functionalization of the thionine molecule.

### Thionine Functionalization

**Figure 1:**
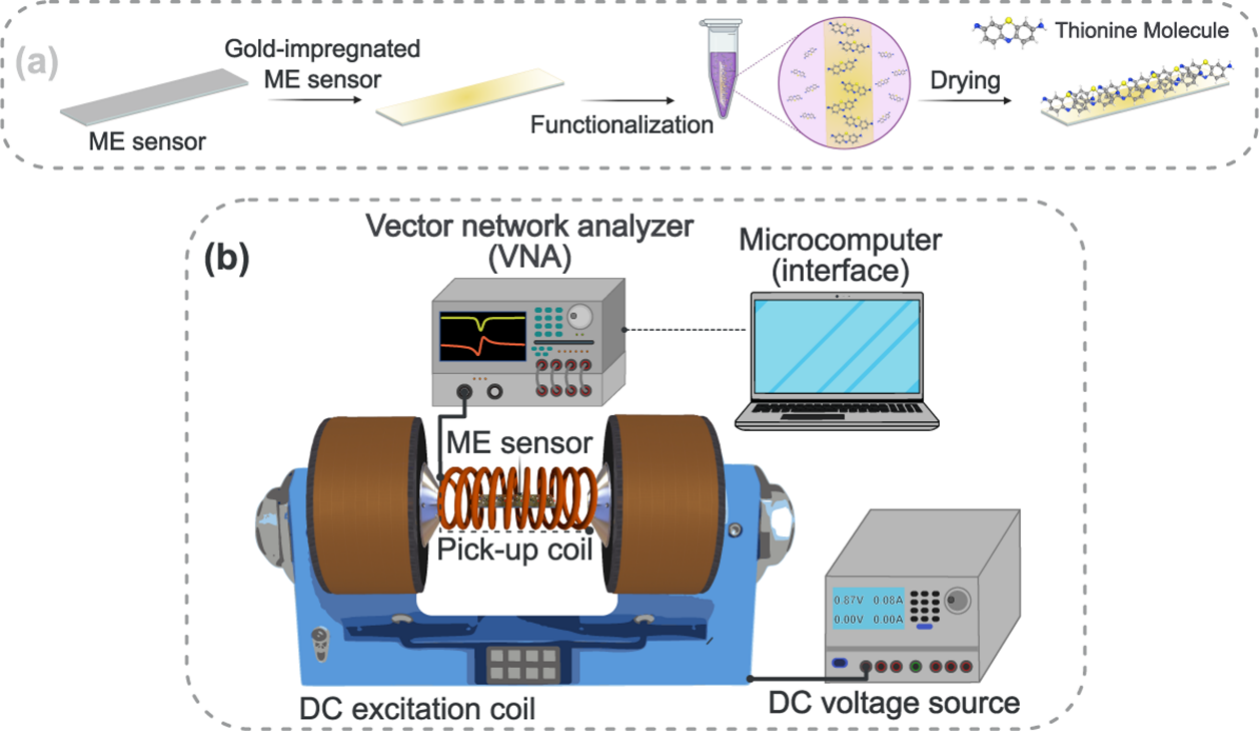
Scheme representing the functionalization steps of the ME sensor (a) and (b) the experimental configuration for measuring magnetoelastic resonance.

The functionalization of the thionine molecule on the Au surface of the ME sensor was carried out by adsorption, inserting the sensor in a thionine chloride solution with a concentration of 0.52 mM for pre-established times (20, 40, 80, and 120 min). In solution, chloride is released from the thionine molecule, which is free to deposit on the Au surface.^14,16^ After functionalization, each sensor is then dried in a controlled nitrogen flux. Finally, the sensor is taken to the magnetoelastic resonance system to measure the ME frequency. The scheme in Figure 1a exemplifies the functionalization process.

### Surface Functionalization Validation

In order to confirm that the thionine molecule was adequately adsorbed on the sensor surface, SEM, KPFM, and micro-Raman spectroscopy were carried out. SEM analysis was performed at an accelerating voltage of 5 kV using a JEOL-JSM-6010LA microscope (JEOL Corporation, Tokyo, Japan) equipped with a silicon-drift detector for EDX characterization. KPFM measurements were performed using a FlexAFM Nanosurf microscope operating in a controlled relative humidity (RH) environment, provided by a hermetic chamber with an N_2_ gas flux. The KPFM signal and topography data were recorded simultaneously in a single scan method via two independent lock-in amplifier channels, each driven at different frequencies. An electrically conductive tip coating with a 5-nm chromium layer and a 25-nm platinum layer on both sides of the cantilever, (ElectricMulti75-G) with a resonance frequency of 75 kHz and a force constant of 3 N/m, was used. The modulation electrical signal was set to an amplitude of 3 V and a frequency of 17 kHz to induce an oscillating electrostatic force between the AFM probe and the sample surface, which allowed for investigations of the potential distribution mapping over the sensor surface. The micro-Raman spectroscopy measurements were performed in an InVia Renishaw spectrometer (Renishaw, Watton-Under-Edge, United Kingdom) using an argon laser (514.5 nm) excitation laser line, focused by a 50× objective lens with a numerical aperture of 0.75. All measurements were collected with a 20-s acquisition time and 6 spectral accumulations. The spectra were deconvoluted using Fityk software.^21^

## Results and Discussion

### Principle of Detection and Resonance Frequency Measurements of ME Biosensors

A magnetic plane-wave-like disturbance is applied to the ME sensor through external magnetic fields *H* = *H*_DC_*+ h*_AC_(ω), where *H*_DC_ and *h*_AC_(ω) are, respectively, the constant and the alternating components, with ω the angular frequency of the AC component. As the ME sensor is subjected to an alternating magnetic field *h*_AC_, it vibrates at the sensor’s magnetoelastic resonance frequency when *ω = ω*_0_, which corresponds to the natural angular frequency of the sensor’s vibration, expressed as follows:
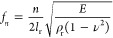
1where *l*_r_ is the sensor length and *f*_*n*_ is the frequency of the ME sensor associated with the vibrational mode *n*. All measurements performed in this work were conducted with *n* = 1, hence *f*_1_ ≡ *f*_0_, with *f*_0_ being the natural resonance frequency of the ME sensor.

By considering a layer of mass uniformly distributed on the ME sensor surface, rigidly deposited and uniformly distributed over its surface, it is possible to treat the layer of mass as part of the sensor. For a thin mass layer, it is assumed that *ρ*_m_*h* ≪ *ρ*_r_*d*, where *ρ*_m_ and *h* are the density and thickness of the deposited mass layer. Therefore, the frequency shift Δ*f* = *f*_0_ – *f* and the quantity *ρ*_m_*h* associated with the deposited mass layer are expressed as follows:^22^
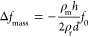
2where Δ*f*_mass_ is the shift in the natural frequency of the ME sensor resulting from the deposition of a thin film on its surface.

For a deposition that occurs randomly across the surface of the ME sensor, there will be a mass distribution σ(*x,y,z*) over the entire area *A*, such that the total mass can be obtained as follows:
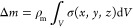
3

Resonance frequency measurements of the ME biosensors were performed using a vector network analyzer (R&SZNLE18, Rohde & Schwarz, Munich, Germany), operated in S_11_ mode and connected to a double-layer excitation/pickup solenoid coil (200 turns, 4 mm in internal diameter and 22 mm in length), which generates the AC excitation signal with a 5 Hz step to perform a frequency sweep and monitor the reflected signal. The DC magnetic induction field was applied through a second solenoid coil (600 turns, 23 mm internal diameter, and 50 mm long, with 0.40 mm thick copper wire and 10.7 ohms resistance) concentric to the excitation/capture coil, using a Keysight U8031A DC source. The reading is performed with the sensor inside an empty 0.5 mL Eppendorf microtube, which is inserted vertically into the excitation/capture coil to measure the magnetoelastic resonance frequency. All measurements were performed at room temperature (25 °C). The resonant frequency of the biosensor is determined by measuring the parameter S_11_. The scheme in Figure 1b depicts the experimental setup used.

**Figure 2:**
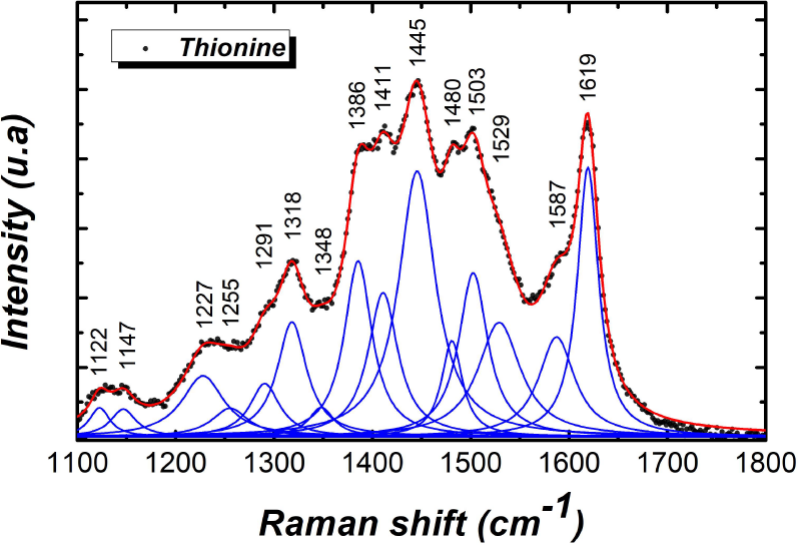
Deconvolution of the Raman spectrum of the thionine-functionalized sensor surface.

Figure 2 shows the Raman spectrum in the range of 1100–1800 cm^–1^ of the functionalized sensor surface. The presence of all Raman peaks assigned to the chemical bonds of thionine molecules confirms the successful functionalization prior to the magnetoelastic characterization.^23−25^

**Figure 3:**
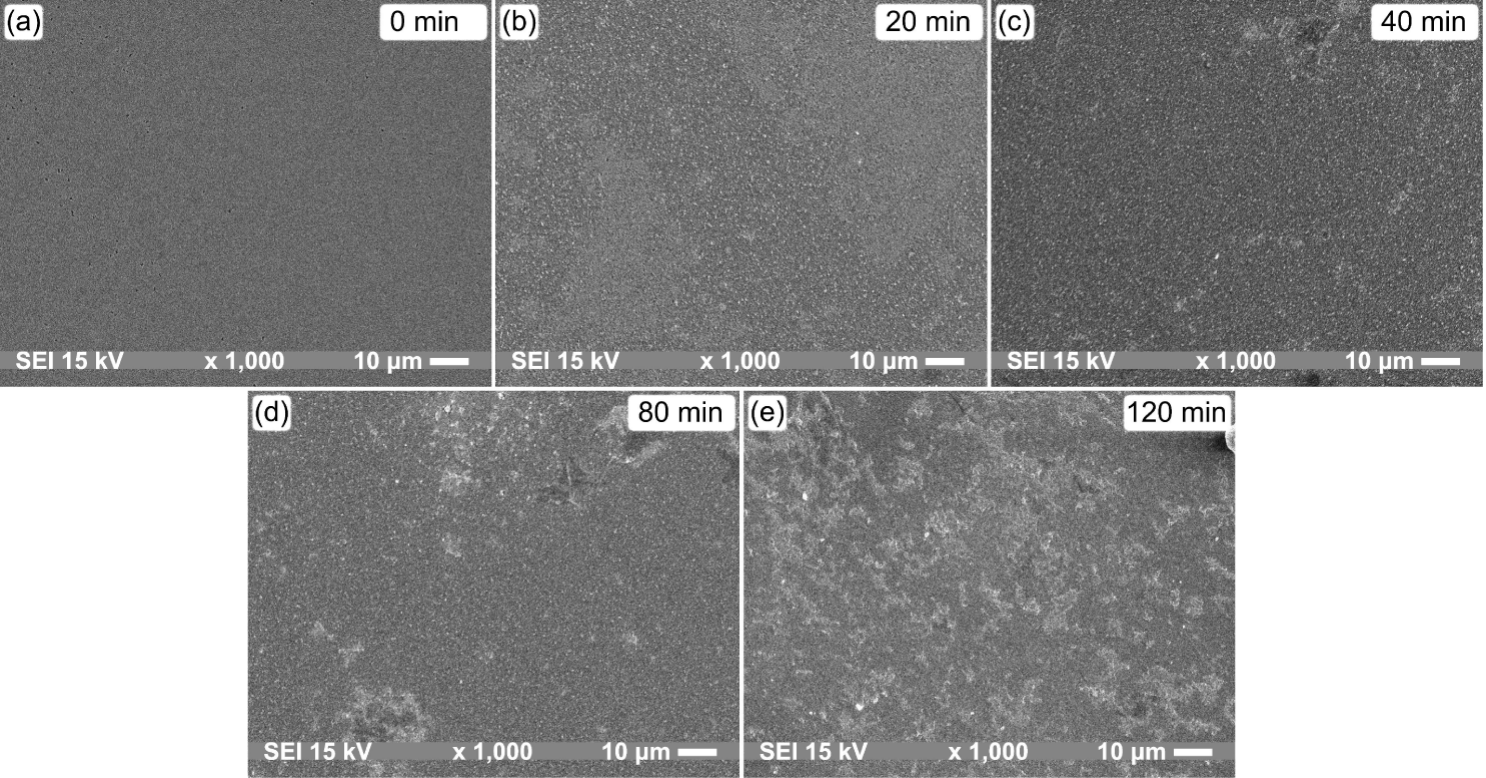
SEM images of the sensor surface for different functionalization times. A formation of clusters of thionine molecules is noticed after 40 min (c), which become more pronounced at 80 and 120 min (d, e).

The images in Figure 3a–e show SEM micrographs of ME sensors with areas of 132.5 μm × 100.0 μm coated by thionine molecules at different functionalization times. Figure 3b shows the deposited layer after 20 min of functionalization, where it is possible to observe the beginning of the aggregation process of thionine covering the Au surface. After 40 min, in the micrograph of Figure 3c, clusters begin to form, distributing throughout the sensor area as the functionalization time increases, with the largest concentration of clusters observed at 120 min.

**Figure 4:**
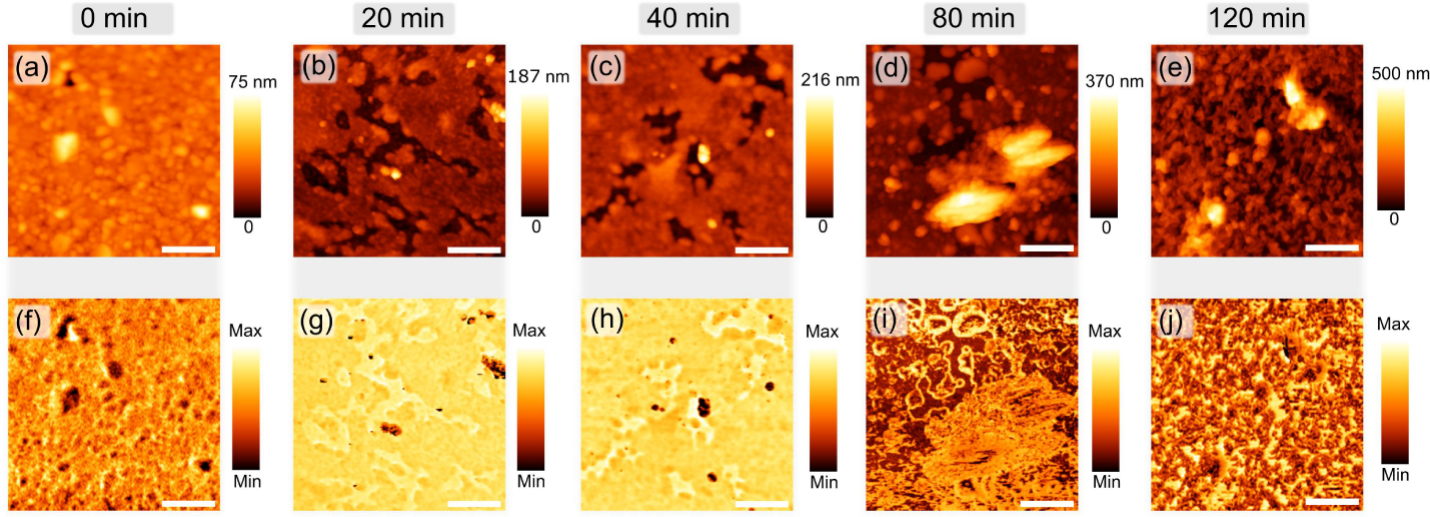
(a–e) Topography and (f–j) corresponding KPFM surface potential images for thionine-gold system as a function of functionalization time. In all images, white scale bars represent 1 μm.

To study the surface properties of the thionine aggregation process on the Au surface, we performed amplitude-modulated KPFM measurements in single-pass scan mode using a FlexAFM (Nanosurf) system. In this imaging mode, one obtains both concomitant topography and surface potential response. Figure 4 (upper panel) shows the topography of the thionine-Au system for different functionalization times, i.e., (a) 0 min, (b) 20 min, (c) 40 min, (d) 80 min, and (e) 120 min. It is interesting to note that 20 min (Figure 4b) of functionalization is sufficient for thionine to cover around 90% of the gold surface, considering several measurements in areas of 5 × 5 μm^2^ (see Figure S2 for the image processing used to evaluate the covered area fraction). In fact, as the functionalization time increases from 20 to 120 min, it is possible to observe a progressive increase in the surface coverage of thionine on the Au. Particularly after 120 min of functionalization, the Au surface is entirely covered by the thionine molecules, forming a uniform molecular film. Nevertheless, the formation of the molecular film presents two distinct regimes depending on the functionalization time. For functionalization times of up to 40 min, it is possible to observe a gradual increase in the surface coverage area of thionine on the gold surface without the formation of large areas with clusters of thionine molecules. On the other hand, for functionalization times longer than 80 min, it is already possible to observe the formation of large clusters of molecules on the thionine film (bright regions in Figure 4c) in good agreement with SEM results.

The KPFM signal (bottom panel of Figure 4) follows the changes in the surface morphology of the thionine-Au system as a function of functionalization time. In organic materials, KPFM measures the contact potential difference, a surface electrical property that is related to a generalized form of the work function (existent in metals). Such capability allows us to understand the evolution of surface electric/electronic conditions for active use in devices. In Figure 4f,g, it is possible to observe the contrast between thionine and gold surface for 20 and 40 min of functionalization time, respectively. The thionine adsorption onto an Au surface slightly modifies the electronic conditions near the gold surface, altering the local electron density and resulting in changes in the CPD. The presence of small molecule clusters is associated with regions where the CPD decreases, suggesting that local molecular orientation restricts the formation of regions with well-differentiated CPD from the Au substrate, probably due to electron hopping. However, the surface potential signal exhibits different features when functionalization time increases above 40 min, as shown in Figure 4g,h for 80 and 120 min, respectively. As a result, we may infer that after 80 min, the dynamics of molecular aggregation begin to change, resulting in a more disordered aggregation, as evidenced in the 120-min sample. We infer that, as the Au surface is covered, the thionine–thionine interactions become favorable, and the KPFM signal is dominated by the existence of large clusters along the thionine film with characteristic CPDs that differ from Au. Such a condition results in additional changes in the surface potentials around thionine cluster edges and domain frontiers. In these locations, observed in brighter color codes in Figure 4i, j, the electronic behavior is greatly affected, qualitatively linking structural changes to the onset of modified electronic responses. This overall electronic behavior has been previously observed in different molecular systems, where surface potential strongly depends on the molecular orientation at the surfaces, the molecular packing density, and the presence of defects in the film.^26^

**Figure 5:**
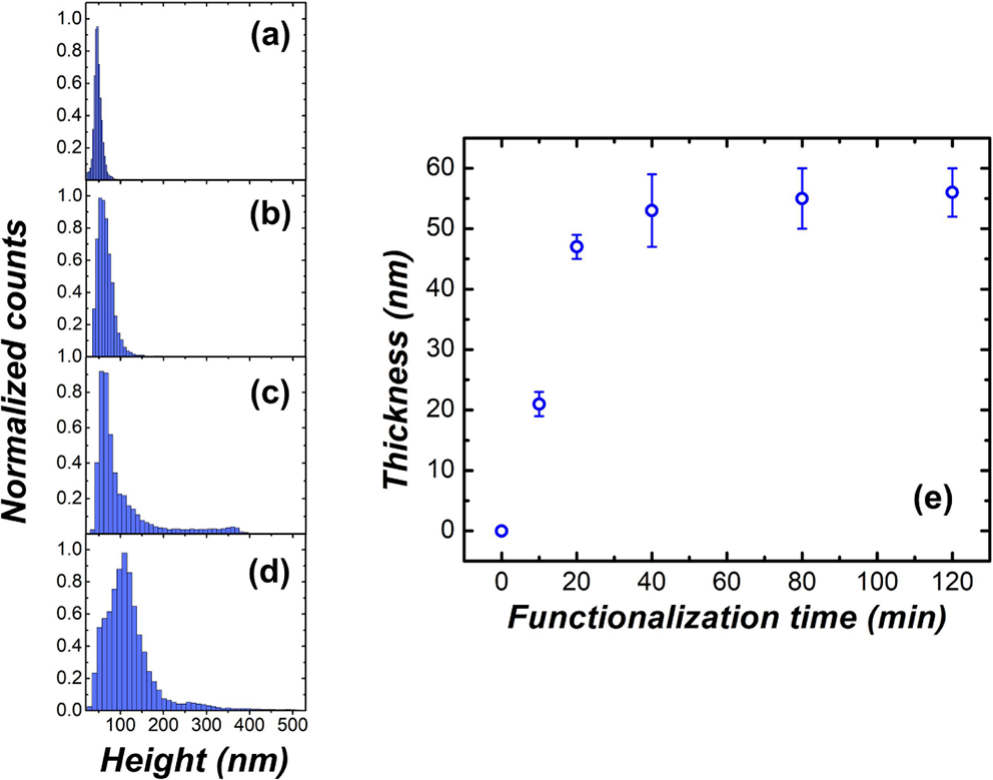
Height distributions of thionine obtained from AFM images over a 5 × 5 μm^2^ area, corresponding to functionalization times of (a) 20 min, (b) 40, (c) 80, and (d) 120 min. (e) Thionine film thickness as a function of functionalization time (not considering large clusters).

The onset of molecular cluster formation can be directly correlated to the thionine saturation of the surface coverage, since the thionine molecules tend primarily to interact with Au atoms.^20^ Indeed, as the functionalization time increases, fewer uncovered gold regions are available for thionine aggregation, resulting in the formation of large regions of molecular clusters for higher functionalization times. Furthermore, considering regions without thionine clusters, the molecular film thickness (see Figure S3 for the image processing methodology used to evaluate the film thickness) tends to saturate at approximately 60 nm as the functionalization time exceeds 40 min, as shown in Figure 5e. This indicates that the gradual variation of surface potentials presented in Figure 4f–j can be associated with mass transport during the functionalization process. For comparison, the evolution of the overall thionine height distribution (including clusters) as a function of functionalization is depicted in Figure 5a–d. For 20 min of functionalization time (Figure 5a), the height distribution exhibits a Gaussian-like profile, indicating a more homogeneous thionine incorporation process. However, as the functionalization time increases (Figure 5b,c) the height distribution begins to deviate from a Gaussian-like behavior, which can be associated with the contribution of thionine cluster formation distributed along the Au surface.

**Figure 6:**
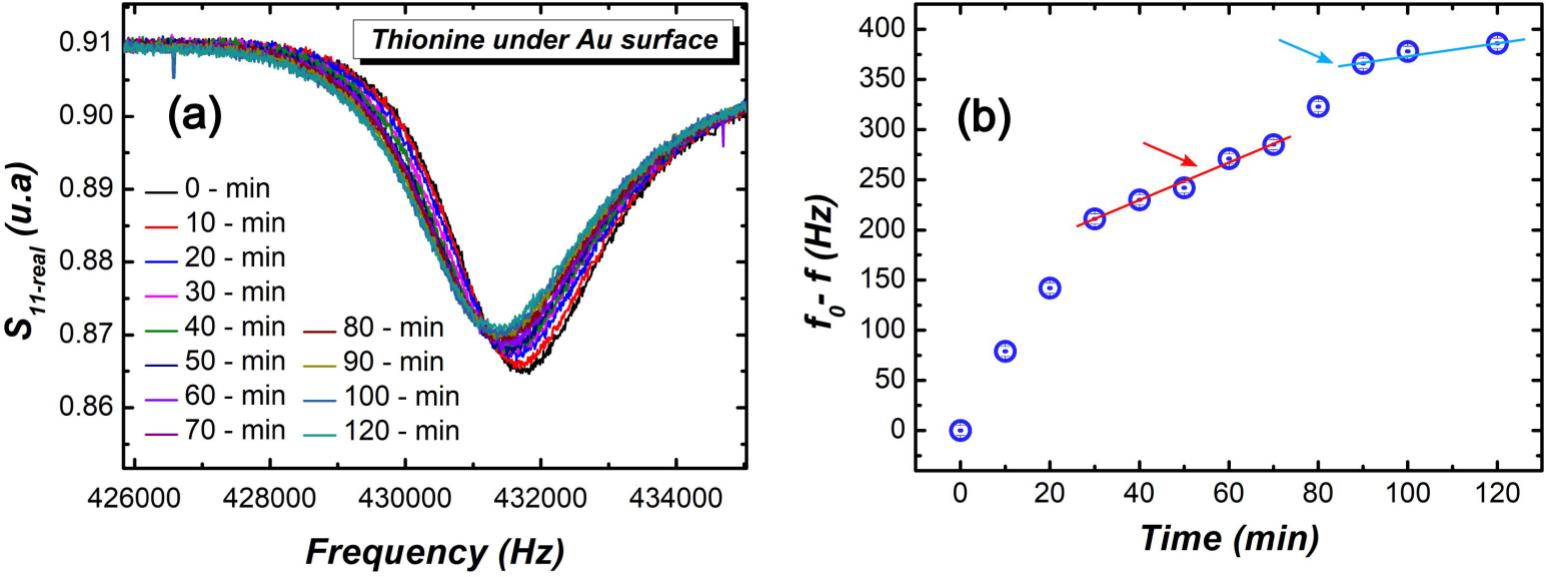
Magnetoelastic resonance spectra (a) for the ME sensor obtained at different functionalization times. In (b), changes in the resonance frequency of the exposed sensor are measured with respect to the sensor without thionine deposition.

The results obtained from the real part of the measurements of the S_11_ parameter express the ME sensor resonance frequency, as presented in Figure 6a. As shown in this figure, the ME sensor resonance frequency decreases as the functionalization time increases due to the incorporation of thionine molecules. Figure 6b shows the resonance frequency shift (Δ*f* = *f*_0_ – *f*) as a function of the functionalization time. A saturation in Δ*f* tends to occur as the time approaches 120 min.

Two regimes were observed in the curve of the magnetoelastic frequency shift, Δ*f*, exhibiting a saturation trend. They are highlighted by the red and blue arrows in the graph of Figure 6b. The first stage, indicated by the red arrow, may result from the surface coverage saturation of thionine atop the sensor prior to the formation of clusters. The graph in Figure 5e supports this hypothesis, as after approximately 40 min, the initial surface coverage reaches saturation, indicating that the mass deposition on the sensor surface becomes progressively smaller. The blue arrow range, where the behavior of the frequency shift of the ME sensor begins to rise again, may be related to an increase in mass on the sensor’s surface due to cluster formation, as indicated in the graphs of Figure 5c,d, which show the height distribution of the deposits on the sensor surface. It can be observed that larger structures emerge, which may lead to an additional disturbance in the sensor response, resulting in a larger shift in Δ*f*, followed by subsequent saturation. This may indicate a decrease in cluster formation.

The difference between the time for Δ*f* saturation and the time for thionine-layer thickness saturation can be explained by the progressive weakening of the thionine layer interaction as the functionalization time increases. Indeed, the gold–thionine interaction is stronger than the thionine–thionine interaction, and the shielding effect increases as the thionine layer thickness grows.^27^ This leads to the instability of the thionine clusters formed on the sensor’s gold surface, as subsequent layers would exhibit reduced adhesion due to the shielding of the initially deposited molecules.^24^ Thionine binds to the Au surface through covalent bonds, specifically via functional groups such as amines, where the -NH_2_ group attaches to the gold surfaces, with the lone electron pair of the nitrogen atom pointing toward the surface, forming a stable layer over the gold.^28,29^ Consequently, after a certain functionalization time, the thionine molecules no longer attach firmly to the sensor surface, explaining the observed saturation trend in Δ*f* after 120 min. The magnetoelastic resonance measurement process enabled the correlation between thionine cluster formation and Δ*f* shifts, highlighting the precision and excellent performance of the ME biosensor in detecting structural changes and the dynamics of substance deposition.

## Conclusion

The functionalization of ME sensors with thionine molecules significantly affects their resonance frequency, with cluster formation playing a crucial role in the sensor’s response. This study offers insights into the adsorption process of thionine on the sensor’s gold surface and its consequences on device performance. Future work can be carried out on modeling and analysis of the heterogeneous distribution of mass on the sensor surface. Such information can be combined with further exploration of measurement strategies, such as AFM, by increasing the analyzed surface area.

## Supporting Information Available

Figure S1: X-ray diffraction data; Figure S2: atomic force microscopy image of thionine-Au system functionalized; Figure S3: AFM image of thionine-gold system functionalized (PDF)
