# Supplementary material for: Investigation of the Impact of Thionine Functionalization on Magnetoelastic Sensor Performance
Source: ACS Appl Bio Mater. 2025 Jan 29;8(3):1997–2002. doi: 10.1021/acsabm.4c01488 (PMC11920945; doi:10.1021/acsabm.4c01488)
Supplement: Supplementary file 1 — mt4c01488_si_001.pdf [file mt4c01488_si_001.pdf]

Supporting information

Investigation of the impact of thionine functionalization on magnetoelastic sensor performance

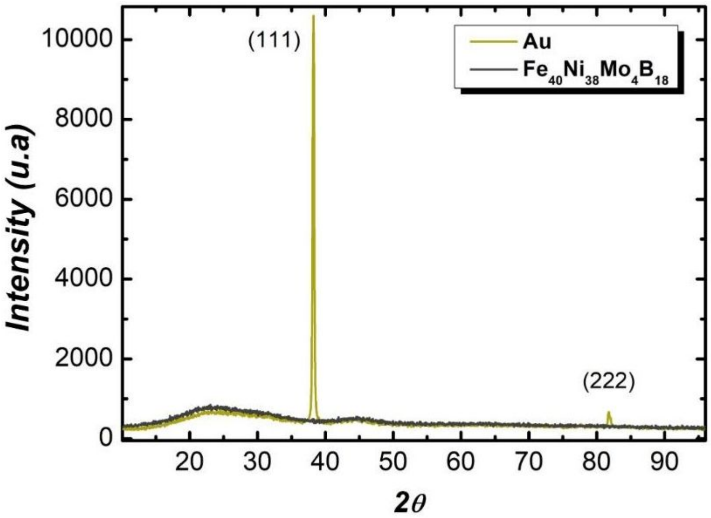

Figure S1 - X-ray diffraction data showing the deposition of 100 nm thick layer of crystalline gold (Au) on METGLAS® 2826MB3 alloy ($Fe_{40}Ni_{38}Mo_4B_{18}$).

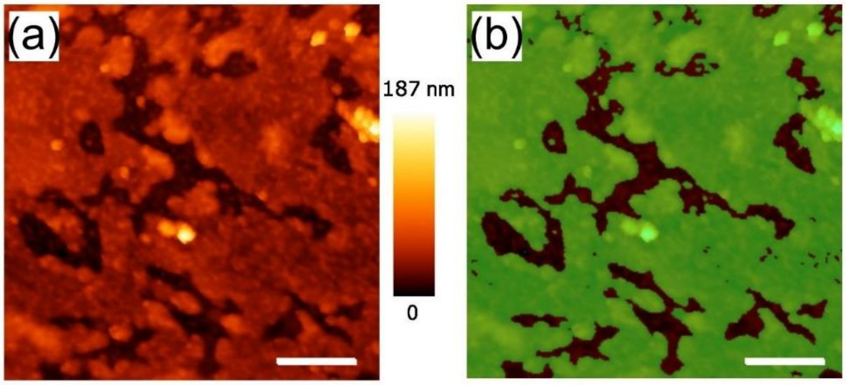

Figure S2 - (a) Atomic force microscopy image of thionine-Au system functionalized during 20 min. (b) Representation of the mask used to estimate the percentage surface coverage of thionine molecules on Au surface. In all images, white scale bars represent 1 μm.

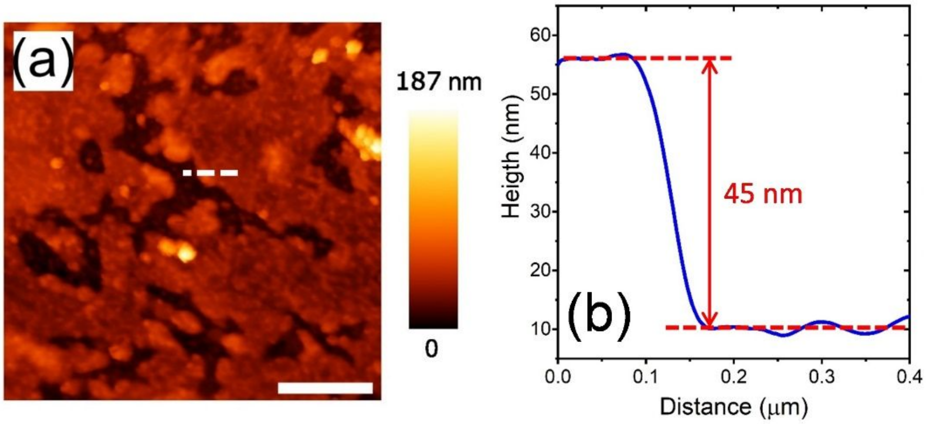

Figure S3 - (a) AFM image of thionine-gold system functionalized during 20 min and (b) corresponding height profile along the white dashed lines of the AFM image. The white scale bar corresponds to 1um.
